# A Phylogeny-Based Global Nomenclature System and Automated Annotation Tool for H1 Hemagglutinin Genes from Swine Influenza A Viruses

**DOI:** 10.1128/mSphere.00275-16

**Published:** 2016-12-14

**Authors:** Tavis K. Anderson, Catherine A. Macken, Nicola S. Lewis, Richard H. Scheuermann, Kristien Van Reeth, Ian H. Brown, Sabrina L. Swenson, Gaëlle Simon, Takehiko Saito, Yohannes Berhane, Janice Ciacci-Zanella, Ariel Pereda, C. Todd Davis, Ruben O. Donis, Richard J. Webby, Amy L. Vincent

**Affiliations:** a Virus and Prion Research Unit, National Animal Disease Center, USDA-ARS, Ames, Iowa, USA; b Bioinformatics Institute, University of Auckland, Auckland, New Zealand; c Department of Zoology, University of Cambridge, Cambridge, United Kingdom; d J. Craig Venter Institute, La Jolla, California, USA; e Department of Pathology, University of California, San Diego, California, USA; f Laboratory of Virology, Faculty of Veterinary Medicine, Ghent University, Merelbeke, Belgium; g Animal and Plant Health Agency, Weybridge, United Kingdom; h National Veterinary Services Laboratories, USDA-APHIS, Ames, Iowa, USA; i ANSES, Ploufragan-Plouzané Laboratory, Swine Virology Immunology Unit, Ploufragan, France; j Division of Transboundary Animal Disease, National Institute of Animal Health, National Agriculture and Food Research Organization, Ibaraki, Japan; k Canadian Food Inspection Agency, National Centre for Foreign Animal Disease, Winnipeg, Manitoba, Canada; l Embrapa Swine and Poultry, Animal Health and Genetic Laboratory, Concórdia, SC, Brazil; m Instituto de Patobiología, CICVyA INTA, Hurlingham, Buenos Aires, Argentina; n Influenza Division, National Center for Immunization and Respiratory Diseases, Centers for Disease Control and Prevention, Atlanta, Georgia, USA; o Department of Infectious Diseases, St. Jude Children’s Research Hospital, Memphis, Tennessee, USA; Emory University School of Medicine

**Keywords:** H1N1, H1N2, influenza A virus, molecular epidemiology, nomenclature, swine, virus evolution

## Abstract

The H1 subtype of influenza A viruses (IAVs) has been circulating in swine since the 1918 human influenza pandemic. Over time, and aided by further introductions from nonswine hosts, swine H1 viruses have diversified into three genetic lineages. Due to limited global data, these H1 lineages were named based on colloquial context, leading to a proliferation of inconsistent regional naming conventions. In this study, we propose rigorous phylogenetic criteria to establish a globally consistent nomenclature of swine H1 virus hemagglutinin (HA) evolution. These criteria applied to a data set of 7,070 H1 HA sequences led to 28 distinct clades as the basis for the nomenclature. We developed and implemented a web-accessible annotation tool that can assign these biologically informative categories to new sequence data. The annotation tool assigned the combined data set of 7,070 H1 sequences to the correct clade more than 99% of the time. Our analyses indicated that 87% of the swine H1 viruses from 2010 to the present had HAs that belonged to 7 contemporary cocirculating clades. Our nomenclature and web-accessible classification tool provide an accurate method for researchers, diagnosticians, and health officials to assign clade designations to HA sequences. The tool can be updated readily to track evolving nomenclature as new clades emerge, ensuring continued relevance. A common global nomenclature facilitates comparisons of IAVs infecting humans and pigs, within and between regions, and can provide insight into the diversity of swine H1 influenza virus and its impact on vaccine strain selection, diagnostic reagents, and test performance, thereby simplifying communication of such data.

**IMPORTANCE** A fundamental goal in the biological sciences is the definition of groups of organisms based on evolutionary history and the naming of those groups. For influenza A viruses (IAVs) in swine, understanding the hemagglutinin (HA) genetic lineage of a circulating strain aids in vaccine antigen selection and allows for inferences about vaccine efficacy. Previous reporting of H1 virus HA in swine relied on colloquial names, frequently with incriminating and stigmatizing geographic toponyms, making comparisons between studies challenging. To overcome this, we developed an adaptable nomenclature using measurable criteria for historical and contemporary evolutionary patterns of H1 global swine IAVs. We also developed a web-accessible tool that classifies viruses according to this nomenclature. This classification system will aid agricultural production and pandemic preparedness through the identification of important changes in swine IAVs and provides terminology enabling discussion of swine IAVs in a common context among animal and human health initiatives.

## Abstract (precis)

A fundamental goal in the biological sciences is the definition of groups of organisms based on evolutionary history and the naming of those groups. For influenza A viruses (IAVs) in swine, understanding the hemagglutinin (HA) genetic lineage of a circulating strain aids in vaccine antigen selection and allows for inferences about vaccine efficacy. Previous reporting of H1 virus HA in swine relied on colloquial names, frequently with incriminating and stigmatizing geographic toponyms, making comparisons between studies challenging. To overcome this, we developed an adaptable nomenclature using measurable criteria for historical and contemporary evolutionary patterns of H1 global swine IAVs. We also developed a web-accessible tool that classifies viruses according to this nomenclature. This classification system will aid agricultural production and pandemic preparedness through the identification of important changes in swine IAVs and provides terminology enabling discussion of swine IAVs in a common context among animal and human health initiatives.

## INTRODUCTION

Influenza A virus (IAV) is one of the most important respiratory pathogens of swine. Infection causes significant financial losses through decreased production, increased vaccination and treatment cost, and increased mortality through interactions with bacterial and other viral infections (1–3). Additionally, swine IAV is a significant zoonotic pathogen with public health relevance; due to the susceptibility of swine to transient infection with IAVs from different species, novel reassorted and potentially pandemic viruses might emerge in swine and spill over to humans (4). Thus, insights into patterns of swine IAV genetic diversity allow identification of novel viral lineages, provide criteria for rational intervention in swine agriculture, and facilitate public health pandemic preparedness.

The global genetic diversity of swine IAV H1 during the last century is a result of the establishment of IAVs from other species in swine populations and subsequent evolution via antigenic shift and drift (5–8). Broadly, there is continual cocirculation of two dominant H1 subtypes (H1N1 and H1N2), within which there are three major lineages resulting from the separate introductions of genetically and antigenically distinct viruses (9, 10). The first endemic swine IAV lineage originated from the 1918 Spanish flu pandemic, leading to the viruses currently classified as “classical-swine” H1N1 (11). In the late 1990s, the classical-swine viruses reassorted their internal genes with those of a lineage of triple-reassortant H3N2 lineage viruses, leading to a spurt of diversification of the hemagglutinin (HA) genes and new genetic H1 clades within the classical lineage (12–15), including the H1N1 pandemic 2009 viruses (H1N1pdm09) (7, 16). The second endemic swine IAV lineage resulted from the spillover of H1 viruses from wild birds in Europe with subsequent export to Asia. Viruses from this lineage are referred to as Eurasian avian-like (10, 17–19). The third endemic swine IAV lineage resulted from repeated human seasonal IAVs spilling into swine herds and subsequent evolution in pigs. These viruses were first recognized in Europe in the 1990s (20), with independent introductions occurring in North American (21, 22) and South American (23) swine herds.

Within these three major lineages, numerous genetic clades of HA have evolved within specific geographical regions, and naming of these clades has been according to regional systems (Table 1). For example, in the United States, a nomenclature system that grouped viruses into one of seven HA H1 clades using Greek letters was adopted (22, 24, 25). In Europe, the European Surveillance Network for Influenza in Pigs (ESNIP) defined four major HA H1 clades, based on host and/or regional introduction history (26). Contemporary HA H1 genes in Europe have been classified as avian-like swine H1_av_N1 lineage, human-like reassortant swine H1_hu_N2 lineage, or H1N1pdm09 lineage; additionally, classical-swine H1N1 viruses were transiently identified in the 1970s and 1980s. Similarly, IAV in Asia reflects the regional introduction and subsequent evolution and cocirculation of multiple genetic clades of classical-swine H1N1, avian-like H1N1, and human seasonal-like H1N1 and H1N2 viruses (6, 27, 28). However, swine move frequently within and sporadically between countries, and clades of originally geographically restricted viruses can be dispersed globally, rendering geographical and regional clade names uninformative. Importantly, current clade descriptors are divorced from a larger evolutionary context that includes H1 viruses from humans and other host species. Furthermore, metrics for genetic differentiation were only arbitrarily applied. For these reasons, a new, adaptable, universally acceptable nomenclature is needed that can follow the dynamic evolution of swine IAV in a globally comprehensive context, both within swine populations and between swine and other hosts. This nomenclature should provide a common terminology for all regions and describe each of the contemporary virus clades in the context of its evolutionary history.

**TABLE 1:** List of colloquial names (if available) for swine influenza A virus H1 hemagglutinin clades and associated laboratory studies

Clade	Colloquial name	Distribution	Reference(s)
Classical swine lineage			
1A.1	α-H1	Canada, China, Hong Kong, Italy, Japan, Mexico, Thailand, United Kingdom, USA	22
1A.1.1		Canada, Hong Kong, South Korea, Taiwan, USA	
1A.1.2		Thailand	
1A.1.3		China, Hong Kong	
1A.2	β-H1	Mexico, South Korea, USA	22
1A.3		USA	
1A.3.1		Mexico	
1A.3.2	γ-2-H1	Mexico, USA	24
1A.3.3		China, Hong Kong, USA	
1A.3.3.1		China	
1A.3.3.2	H1N1pdm09	37 countries	4, 16
1A.3.3.3	γ-H1	South Korea, USA	22
Human seasonal lineage			
1B.1	European human-like reassortant H1_hu_N2 (derived from A/swine/Scotland/410440/94)	Ireland, United Kingdom	26
1B.1.1		France, United Kingdom	26
1B.1.2		Spain, United Kingdom	26
1B.1.2.1		Belgium, Germany, Italy, Netherlands, Spain	26
1B.1.2.2	A/swine/Italy/4675/2003	Italy	26
1B.1.2.3		France	26
1B.2		Argentina, Chile, China, Hong Kong, Japan, Mexico, USA, Vietnam	22
1B.2.1	δ-2	USA	21
1B.2.2	δ-1	Argentina, Brazil, Canada, United Kingdom, USA	21
1B.2.2.1	δ-1a	USA	
1B.2.2.2	δ-1b	USA	
Eurasian avian lineage			
1C.1	Avian-like swine H1_av_N1 (derived from A/swine/Arnsberg/6554/1979 and A/swine/Belgium/WVL1/1979)	Belgium, Canada, France, Germany, Hong Kong, Ireland, Italy, Netherlands, Spain, United Kingdom	15, 61
1C.2	Avian-like swine H1_av_N1 (derived from A/swine/Ille et Vilaine/1455/1999)	Belgium, Denmark, Finland, Germany, Italy, Mexico, Netherlands, Poland, Sweden	15, 61
1C.2.1		Belgium, Denmark, France, Germany, Hungary, Italy, Netherlands, Poland, Russia, Spain	
1C.2.2		France, Germany, Italy, Luxembourg, Netherlands, Poland, Spain	
1C.2.3		China, Czech Republic, France, Hong Kong, Italy, Poland, South Korea	

Here, we collated and analyzed publicly available swine H1 data from 1933 to 2015 to address this issue. Using a series of objective phylogenetic metrics in concordance with the tacit goals of the WHO/OIE/FAO H5N1 Working Group (29), a unified swine H1 HA nomenclature system was established to simplify terminology, remove the arbitrary association with geography, establish a rational system for identifying and designating future clades, and link the evolutionary history of all swine H1 IAVs with common ancestral lineages. Further, we developed a web-based annotation tool that uses the principles of the proposed nomenclature to assign clade designations to swine HA/H1 sequence data. The tool places an HA/H1 sequence on a phylogeny of just a few representatives of each of the named clades and then infers a clade for the query sequence from its local environment in the phylogeny. Classification by this web-based tool matched expertly curated, manual classification of the sequences >99% of the time. This tool will be released on the Influenza Research Database (IRD) at http://www.fludb.org (30, 31) to facilitate the adoption of the unified nomenclature.

## RESULTS

### Global genetic diversity and swine H1 clade designations.

**FIG 1:**
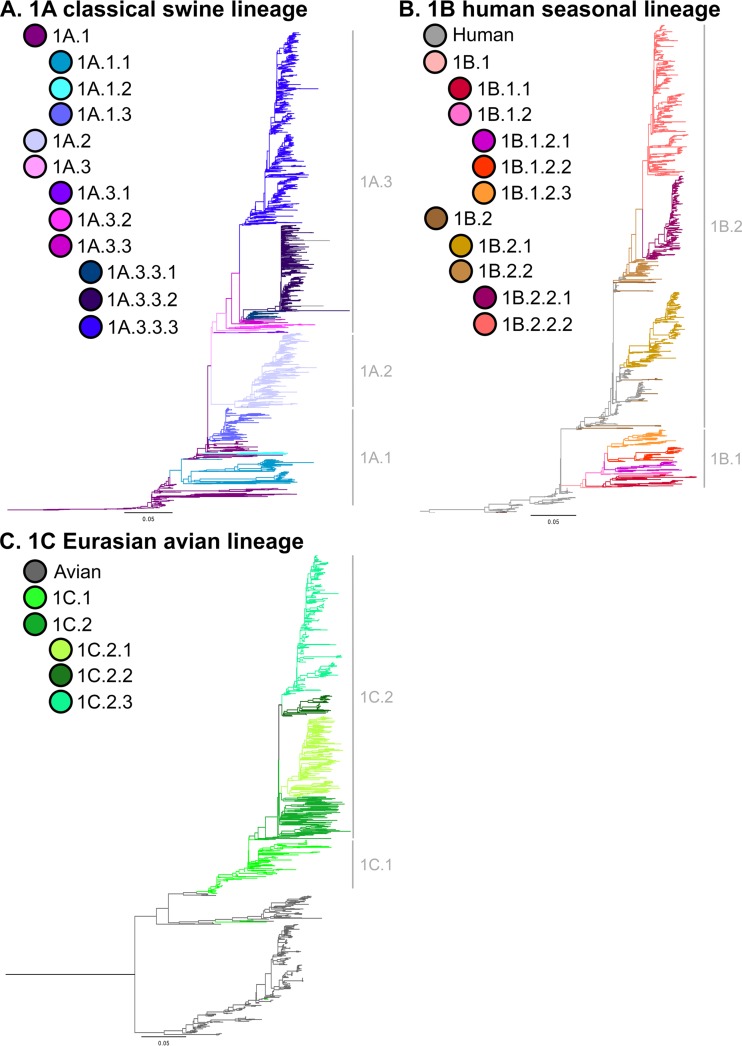
Phylogeny of swine H1 influenza A virus hemagglutinin gene sequences. The best-known tree was generated using maximum likelihood methods from 7,070 H1 swine and representative human and avian hemagglutinin gene sequences. Subsequently, the global tree was split into the three major lineages to facilitate presentation: 1A classical swine lineage (A), 1B human seasonal lineage (B), and 1C Eurasian avian lineage (C). Branch color represents clade designations based on the nomenclature system proposed in this study. Each tree is midpoint rooted for clarity; all branch lengths are drawn to scale, and the scale bar indicates the number of nucleotide substitutions per site. The global phylogeny with bootstrap support values and tip labels is provided in Fig. S1 in the supplemental material.

**FIG 2:**
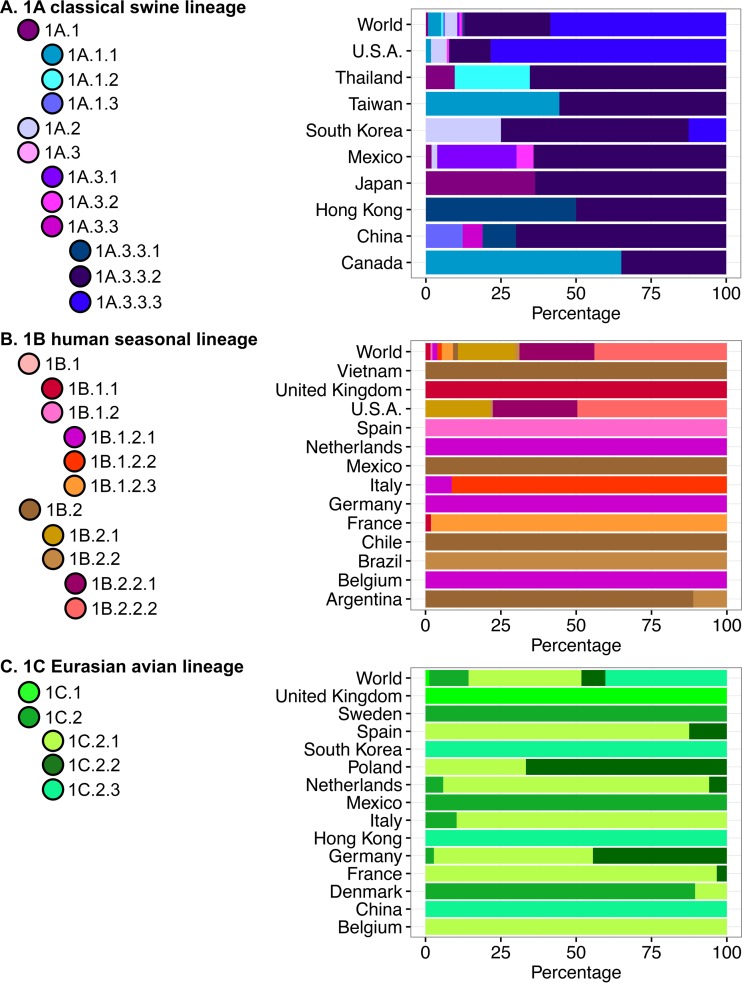
Global distribution of swine H1 influenza A virus hemagglutinin clades from 2010 to present. The three panels reflect the three major lineages: 1A classical swine lineage (A), 1B human seasonal lineage (B), and 1C Eurasian avian lineage (C). The proportion of each H1 HA clade reported from 2010 to present is represented by the color in each country; the “World” designation represents the cumulative proportion for each H1 HA clade across the three lineages. Colors follow those in Fig. 1 and reflect the nomenclature system proposed in this study.

Substantial genetic diversity was demonstrated in H1 viruses circulating in swine over the past 5 years (2010 to present) and among geographic regions (Fig. 1 and 2). Three major first-order H1 lineages continued to circulate in pigs (Fig. 1; also see Fig. S1 in the supplemental material): the 1A classical lineage, viruses related to the 1918 human influenza pandemic; the 1B human seasonal lineage, the result of multiple human-to-swine transmission episodes of human seasonal H1 strains over decades; and the 1C Eurasian avian lineage, arising from an introduction from wild birds into pigs in the 1970s. The majority (~87%) of the viruses from 2010 to the present were placed into seven clades. The numerically dominant clades reflected intensive surveillance in the United States (24, 25), investigator sequencing efforts in Canada (e.g., references 32 and 33), and the rapid dissemination of the 2009 H1N1 pandemic virus (H1N1pdm09) across global swine populations (7, 16). Similarly, coordinated surveillance in Europe (26, 34) and Asia (6) captured two primary clades of 1C Eurasian avian lineage currently circulating in the two continents.

10.1128/mSphere.00275-16.1 Figure S1  Phylogeny of swine H1 influenza A virus hemagglutinin gene sequences. The best-known tree generated using maximum likelihood methods from 7,070 H1 swine and representative human and avian hemagglutinin H1 gene sequences is provided in Nexus tree format. Branch color represents clade designations proposed in this study. The tree is midpoint rooted for clarity; all branch lengths are drawn to scale, and the scale bar indicates the number of nucleotide substitutions per site. Download Figure S1, TXT file, 1.3 MB. Copyright © 2016 Anderson et al. 2016 Anderson et al. This content is distributed under the terms of the Creative Commons Attribution 4.0 International license.

### Clade designations for 1A (classical) swine lineage.

The 1A (classical) lineage contained 1,889 viruses from 34 countries collected from 2010 to the present (Fig. 1 and 2). According to our nomenclature rules, we refined the classification of 1A viruses into three second-order divisions, each of which corresponds to earlier, regional classifications (25): 1A.1 viruses, similar to classical “α-H1” viruses (*n* = 120, 7 countries); 1A.2 viruses, similar to “β-H1” viruses (*n* = 77, from United States, South Korea, and Mexico); and 1A.3 viruses, similar to “γ-H1” viruses (*n* = 1,692, 34 countries). Applying our nomenclature rules again leads to further subdivision of these second-order divisions into third- and fourth-order divisions. The only clade within the 1A lineage that had wide global distribution was that of the 2009 H1N1 pandemic clade (1A.3.3.2) with 541 viruses in 34 countries. Some clades within the 1A lineage were geographically constrained: 1A.1.2 viruses were detected only in Thailand (*n* = 13); 1A.1.3, 1A.3.3, and 1A.3.3.1 were restricted to China and Hong Kong (*n* = 28); and 1A.3.1 was restricted to Mexico (*n* = 14). The remaining clades were more diffuse, potentially reflecting the dissemination of viruses with agricultural trade (see reference 35). For example, the 1A.1.1 clade was detected in Canada, the United States, and Taiwan (*n* = 82); the 1A.3.2 clade was detected in Mexico and the United States (analogous to “γ-2 H1” viruses [24]); and the 1A.3.3.3 viruses were isolated in South Korea and the United States (analogous to “γ-H1” viruses).

The 1A classical lineage within- and between-clade average pairwise distances (APDs) are presented in Table 2. Each clade had an APD of >7% from other clades and an APD of <7% within the clade, although some minor exceptions were made when all other clade-defining criteria were met and mitigating circumstances supported the exception. Within-clade exceptions were made for the first-order 1A.1 (APD, 7.8%) and the extensive 1A.1.1 second-order clade (APD, 9.5%) that represented multiple monophyletic clades of viruses that individually did not meet our criteria for further division based on the number of recent sequences. The exception to the >7% distance between-clade threshold was associated with clades nested within 1A.3.3 (1A.3.3.1, 1A.3.3.2, and 1A.3.3.3); however, the 1A.3.3 clade required these additional third-order divisions to differentiate the H1N1 pandemic viruses (1A.3.3.2) from North American “γ-H1” viruses (1A.3.3.3) and a geographically isolated clade of viruses from China (1A.3.3.1).

**TABLE 2:** Average percent pairwise nucleotide distances within and between H1 1A (classical lineage) phylogenetic clades

Clade	APD (%)											
	Within clade	Between clade:										
		1A.1	1A.1.1	1A.1.2	1A.1.3	1A.2	1A.3	1A.3.1	1A.3.2	1A.3.3	1A.3.3.1	1A.3.3.2
1A.1	7.8											
1A.1.1	9.5	11.5										
1A.1.2	5.4	11.4	13.8									
1A.1.3	3.0	8.3	11.8	10.1								
1A.2	3.8	10.8	14.0	12.5	8.7							
1A.3	4.2	10.8	13.7	12.2	8.7	10.6						
1A.3.1	1.4	11.2	13.7	12.7	9.1	12.1	11.4					
1A.3.2	5.3	11.1	14.1	12.7	9.0	11.3	10.0	11.2				
1A.3.3	5.1	10.1	13.4	11.6	7.6	10.2	9.7	10.2	8.1			
1A.3.3.1	2.7	10.5	13.5	12.1	7.9	10.1	10.0	10.3	8.3	5.0		
1A.3.3.2	1.9	12.0	14.4	13.5	9.7	11.1	11.3	12.0	9.8	7.5	6.5	
1A.3.3.3	3.2	11.1	14.1	12.5	9.0	10.7	10.9	10.9	9.0	6.8	6.5	8.3

### Clade designations for 1B (human seasonal) swine lineage.

The 1B (human seasonal) lineage contained 1,447 viruses from 13 countries collected from 2010 to the present (Fig. 1 and 2). Applying our nomenclature rules led to two second-order divisions corresponding to established clades: the 1B.1 viruses, related to a reassortant H1N2 virus that emerged in Great Britain in 1994 (*n* = 132, 7 European countries) (20), and 1B.2 viruses, related to the “δ-1 H1” and “δ-2 H1” viruses (*n* = 1,315, 6 countries) (22). We defined two third-order 1B.1 clades: the 1B.1.1 (*n* = 24) viruses, circulating predominantly in the United Kingdom, with one virus collected in France, and 1B.1.2 (*n* = 108) viruses circulating in continental Europe. The fourth-order divisions of 1B.1.2 reflect geographic boundaries: 1B.1.2.1 (*n* = 24) from Belgium, Germany, Italy, and Netherlands; 1B.1.2.2 (*n* = 21) from Italy; and 1B.1.2.3 (*n* = 54) from France.

The 1B.2 clade contained two third-order clades that corresponded to previously described “δ-2 H1” (1B.2.1) and “δ-1 H1” (1B.2.2) clades. Based on average pairwise distances, and a large number of viruses, the third-order 1B.2.2 clade met the criteria for further subdivision into 1B.2.2.1 (*n* = 360) and 1B.2.2.2 (*n* = 636). In addition to these named subdivisions, the 1B.2 clade from 2010 to the present contained sporadic human-to-swine transmission episodes (*n* = 7) in Argentina, Chile (36), China, Mexico, and Vietnam; these spillovers did not warrant the designation of a clade either due to failure to establish in swine populations or due to insufficient numbers to meet our criteria. Similarly, 1B.2.2 (22) included viruses collected from spatially isolated swine populations in Argentina and Brazil (23) and in Mexico that represent human-to-swine transmission episodes, but the number of viruses is too low to be able to confidently infer a separate clade. To link these viruses to their source population and maintain flexibility should additional surveillance detect more samples, we classified these viruses as “Other-Human.”

The 1B human seasonal lineage within- and between-clade APDs are presented in Table 3. For the most part, each clade had an APD of >7% from other clades and almost all had an APD of <7% within the clade. The within-clade exceptions were the 1B.1 and 1B.2 clades (APD, 9.9% and 7.5%, respectively). The 1B.1 second-order clade (*n* = 5) had too few representative sequences to calculate genetic distance, and 1B.2 represented multiple monophyletic clades that individually did not meet our criteria for further division. Similarly, the extensive 1B.1.1 clade (APD, 7.8%) did not meet criteria for further splitting. The exception to the between-clade threshold was associated with clades nested within 1B.2.2 (1B.2.2.1 and 1B.2.2.2). These third-order clade designations were made because of the considerable number of viruses in 1B.2.2 (*n* = 1,016 from 2010 to present), strong bootstrap support (100%), and moderate between-clade support (APDs of 6.4% and 5.8%, respectively).

**TABLE 3:** Average percent pairwise nucleotide distances within and between H1 1B (human seasonal lineage) phylogenetic clades

Clade	APD (%)										
	Within clade	Between clade:									
		1B.1	1B.1.1	1B.1.2	1B.1.2.1	1B.1.2.2	1B.1.2.3	1B.2	1B.2.1	1B.2.2	1B.2.2.1
1B.1	9.9										
1B.1.1	7.8	12.3									
1B.1.2	5.3	12.0	12.2								
1B.1.2.1	5.3	12.4	12.1	9.8							
1B.1.2.2	4.4	13.1	12.6	10.1	9.9						
1B.1.2.3	5.1	12.1	11.8	9.1	9.2	9.0					
1B.2	7.5	12.6	13.5	13.7	13.8	14.7	13.4				
1B.2.1	4.2	14.5	15.1	15.4	15.7	16.6	15.2	8.8			
1B.2.2	3.5	13.9	14.2	14.6	14.6	15.4	14.0	8.1	8.7		
1B.2.2.1	2.3	14.6	15.1	15.6	15.8	16.1	15.2	9.7	10.5	6.4	
1B.2.2.2	2.8	14.1	14.5	14.8	15.0	15.3	14.3	9.1	9.9	5.8	5.3

### Clade designations for 1C (Eurasian avian) swine lineage.

The 1C (Eurasian avian) lineage consisted of 315 viruses from 14 countries collected from 2010 to the present (Fig. 1 and 2). During this time period, we identified two second-order divisions of geographically isolated virus clades: the 1C.1 viruses in the United Kingdom and the 1C.2 viruses in continental Europe and Asia. Within the 1C.2 clade, three third-order divisions emerged: 1C.2.1 (*n* = 118) in Belgium, Denmark, France, Germany, Italy, Netherlands, Poland, and Spain; 1C.2.2 (*n* = 25) in France, Germany, Netherlands, Poland, and Spain; and 1C.2.3 (*n* = 127) in China and South Korea. Avian H1 HA sequences were generally restricted to two monophyletic clades distinct from, but sister to, the 1C swine viruses: these HA sequences were defined as “Other-Avian.” The within- and between-clade APDs are presented in Table 4. For the most part, each clade had an APD of >7% from other clades and an APD of <7% within the clade. The one within-clade exception in this lineage was 1C.2 (APD, 7.9%), which had multiple monophyletic subclades without adequate statistical support to further divide the data.

**TABLE 4:** Average percent pairwise nucleotide distances within and between H1 1C (Eurasian avian lineage) phylogenetic clades

Clade	APD (%)				
	Within clade	Between clade:			
		1C.1	1C.2	1C.2.1	1C.2.2
1C.1	6.7				
1C.2	7.9	10.1			
1C.2.1	5.1	10.0	8.0		
1C.2.2	4.7	10.3	8.5	8.0	
1C.2.3	3.3	9.8	7.8	7.3	7.5

### Consistency of proposed classifications.

The clades identified by these global phylogenetic analyses and pairwise-distance criteria were consistently segregated by different phylogenetic approaches and with randomly subsampled data sets. While tree topology varied slightly between Bayesian and maximum likelihood methods, the monophyletic grouping and bootstrap support (or posterior probability) were consistent. There were a number of minor discrepancies in our classification (*n* = 7 or 0.28% of the randomly subsampled 2,528 viruses [see Data Set S1 in the supplemental material]). Of the 7, 1 HA was incorrectly classified (i.e., 1A.2 virus classified to 1A.3), 1 HA was incorrectly assigned to a lower-order division (1A.1 virus was placed in the 1A.1.2 clade), and the remaining 5 viruses were incorrectly assigned to a higher-order division (1A.3.3 classified to 1A.3).

10.1128/mSphere.00275-16.3 Data Set S1  List of NCBI GenBank accession numbers, strain name, year, subtype, host, geographic location, and clade designation for all sequences analyzed in this study. Additional clade classification columns are from randomly subsampled data and automated tool analysis and indicate whether there is a match or not to the manual global classification. Download Data Set S1, XLSX file, 0.5 MB. Copyright © 2016 Anderson et al. 2016 Anderson et al. This content is distributed under the terms of the Creative Commons Attribution 4.0 International license.

### Automated classification of swine H1 hemagglutinin sequences.

The representative phylogeny used for classifying global swine sequences contained 239 H1 viruses of predominantly swine origin, with a few H1 viruses from human and avian hosts to represent the diversity of nonswine H1 viruses. The swine viruses were selected to capture the diversity within each of the defined clades. We used this algorithm to classify all sequences in the final data set of 7,070 IAV HA/H1 sequences from swine, avian, and human hosts, described in Materials and Methods. The classifier ascribed the correct clade in all but 41 instances. Of these 41 sequences, three from clade 1A.3.3.1 were incorrectly assigned clade 1A.3.3. The remaining 38 sequences were assigned a “-like” classification very close to the correct value. For example, five 1A.1 swine sequences were assigned the classification “1A.1-like.” One 1C.2.1 swine sequence was assigned the classification “1C.2-like.” Overall, the classifier was highly accurate in correctly capturing the classifications assigned by the earlier expert phylogenetic curation. Thus, this tool will be valuable for rapidly assigning the appropriate, biologically meaningful clade to new viruses not studied in our analyses. Its implementation on the web, through IRD (http://www.fludb.org), will allow classification of novel sequences to be carried out in clinical or diagnostic settings.

## DISCUSSION

Swine influenza was first observed in 1918, with the ancestral “classical” H1N1 virus isolated from swine in the 1930s. At present, there are three major evolutionary lineages circulating in swine globally, resulting from the 1918 H1N1 human pandemic, human seasonal H1 viruses, and an avian H1 lineage. As lineages of these viruses were established locally, many of them became ecologically isolated, resulting in divergent evolutionary trajectories (15). We identified 3 first-order lineages, 7 second-order divisions, 13 third-order divisions, and 8 fourth-order divisions that sufficiently capture the historical and current genetic diversity of global swine H1 HA influenza viruses. In doing so, we established rational and rigorous criteria for naming such clades. These criteria are flexible enough to adapt to continued within-clade evolution of viruses and allow for the identification and classification of novel lineages should they emerge.

Our primary goal was to classify HA clades that reflected the evolutionary history of swine IAV. To do so, we use three first-order descriptors—the 1A classical lineage derived from the 1918 human pandemic viruses, the 1B human seasonal lineage associated with 1990s human-to-swine transmission episodes, and the 1C Eurasian avian lineage associated with viruses introduced to swine in Europe and Asia from wild birds (37). Following this, we identified monophyletic clades in our phylogeny with at least 10 viruses collected over the preceding 5 years: without exception, these clades had statistical support of ≥70% and generally an average pairwise distance of <7% within clade and >7% between clades. When applying these criteria with different data sets, there were minor discrepancies (*n* = 7): this highlights the nondeterministic nature of maximum likelihood phylogenetic approaches. The solution to this problem is to use multiple approaches, to use more comprehensive data sets, to conduct analyses more than once, and to interpret the data conservatively.

To facilitate the adoption of this system, we implemented an automated annotation tool that can rapidly assign these biologically informative clade designations to new, as-yet-unclassified sequence data. Our tool uses maximum likelihood to rapidly classify a query IAV sequence by placing it on a reference phylogeny of just 239 H1 viruses selected from the named, biologically informative clades. When a query sequence is placed within a named clade, this name is assigned to the query. When a query sequence does not fall within a named clade, it is classified by the neighborhood of its placement, using a “-like” annotation. For example, the tool assigns the classification “1B.2.2-like” to viruses ancestral to both the 1B.2.2.1 and 1B.2.2.2 clades but not placed within the 1B.2.2 clade (see Fig. S2 in the supplemental material). These “-like” viruses have insufficient statistical support to assign them to a monophyletic clade, forcing a placement between existing clades. By using our automated classification, sequences collected during surveillance efforts can quickly be classified to known clades or, if receiving a “-like” designation, can be flagged for additional analyses or additional targeted sample collection.

10.1128/mSphere.00275-16.2 Figure S2  Classification of query sequences. Q1, Q2, Q3, query sequences. Triangles, tip nodes (viruses with known clades). Circles, internal nodes, with assigned clade designations. Q1 attaches to a terminal branch and is assigned clade 1B.2.2. Q2 attaches to an internal branch connecting clades 1B.2.2.1 and 1B.2.2.2. Q2 does not belong to either clade. Therefore, it is conservatively assigned the clade 1B.2.2-like. Q3 is attached to an internal branch connecting clades 1B.2.2-like and 1B.2.2 and is assigned clade 1B.2.2. Assignment of clades to internal nodes is carried out by the classifier according to a custom parameter file developed for the specific application. Download Figure S2, TIF file, 0.2 MB. Copyright © 2016 Anderson et al. 2016 Anderson et al. This content is distributed under the terms of the Creative Commons Attribution 4.0 International license.

Our goal of achieving tightly structured definitions for statistically supported clades was challenged by the relatively frequent introduction of avian and human IAVs to swine populations (e.g., see references 5 and 38) and the absence of surveillance in large sections of the world, including some with significant swine populations (10). Another challenge was the forced inclusion of viruses with likely specific regional evolutionary histories into a geographically broader classification because of the paucity of sequences from that region. For example, a small cluster of distinct human seasonal viruses in Brazil (23) were classified as 1B.2.2 although they differed from other 1B.2.2 viruses that circulate in different geographic regions. A unique clade designation for this handful of Brazil viruses might be considered if phylogenetic support was >70% and if additional evidence demonstrated continued circulation of this genetic grouping, such as specific hemagglutination inhibition serosurveillance data. These modified criteria (high statistical support and serosurveillance data) may be applied to interspecies spillover events and undersampled regions and allow the creation of further meaningful clade divisions when additional virologic sampling and sequencing are not feasible.

The most readily available approach to limiting IAV transmission within swine populations is through an appropriate vaccination program that protects against currently circulating genetic and antigenic diversity (15, 39, 40). Importantly, our global classification scheme can inform vaccine strain selection: it is not possible to compose a vaccine with all known viral variants (41), and our scheme provides a mechanism for quickly filtering data spatially and temporally, allowing matching to existing vaccines or selection of representative viruses for vaccine research and development. Experimental studies have demonstrated that protection against infection may be correlated with genetic relatedness of the vaccine strain to challenge strains (e.g., see references 42 and 43). However, vaccine efficacy would likely be compromised when considering all clade levels because there are a substantial number of viruses belonging to as many as 18 genetic second- and third-order clades in each continent (e.g., the United States has 12 cocirculating H1 genetic clades), genetic relatedness is not always a good predictor of protection (e.g., see reference 44) because just one or two amino acid mutations in the HA-1 domain may drive a significant reduction in antigenic cross-reactivity (e.g., see references 21, 45, and 46), and host immune response affects protection (47–49). Despite this challenge, and in lieu of a universal vaccine, our classification system can identify regional patterns of genetic diversity, which can lead to assessment of antigenic diversity relative to other viruses (15). For example, if the widely dispersed 1A.3.3.2 viruses (*n* = 541, H1N1pdm09 viruses) are excluded, 84% of the publicly available swine H1 viruses from 2010 to the present belonged to 6 predominant clades: one from the 1A classical lineage, three from the 1B human seasonal lineage, and two from the 1C Eurasian avian lineage. Though there is no centralized system for matching circulating strains with vaccine seeds, these data and the relatively slow antigenic drift of swine viruses (average of 0.39 antigenic units per year for classical-lineage viruses [15]) suggest that a selection of viruses with regional representation would be sufficient for an acceptable vaccine efficacy that reduces clinical burden and limits virus spread.

Swine IAV evolution is a complex issue at regional and especially at global levels. The emergence and extinction of clades due to ecological and evolutionary processes, along with spillover events from nonswine hosts, have created a nomenclature quagmire. Consequently, we developed a unified system that accounts for the unique evolutionary history of swine IAV that can be periodically updated as viral diversity expands or contracts. The data to create a classification system and the accompanying automated tool rely exclusively on genetic divergence in the HA and do not infer information on viral phenotype. Future modeling and computational tools can build from and adapt this system. For example, the classification of nonswine H1 viruses could follow the process described here for swine H1, leading to a comprehensive, multihost H1 classification scheme. Incorporating data from functional HA studies could refine clade definitions. For example, including studies on antigenic evolution with genetic classification could provide advanced metrics for clade definition, which would facilitate the selection of vaccine strains and inform risk management policies for agricultural and public health.

## MATERIALS AND METHODS

### Swine influenza A virus hemagglutinin H1 data set.

All available swine IAV hemagglutinin (HA) H1 sequences from viruses in the IRD (30) were downloaded on 7 June 2016. Only H1N1 and H1N2 subtype viruses were included, and these sequences comprised 8,438 worldwide samples. To restrict our analyses to relevant field viruses, we excluded sequences with “lab” or “laboratory” host. Sequences were then aligned with MAFFT v 7.221 (50, 51), with manual correction and curation in Mesquite (52). The aligned sequences underwent a redundancy analysis within the program mothur v.1.36.0 (53), and sequences with 100% identity were removed. Our final filtering step was to remove poor-quality data using two criteria: sequences were removed if >50% of the HA gene sequence was missing and a sequence was removed if it had more than 5 nucleotide base ambiguities. This process resulted in a set of 6,298 nonidentical H1 HA swine IAV sequences that represent the full extent of published swine H1 HA genetic diversity worldwide. An additional 428 randomly sampled human seasonal H1 HA sequences and 344 randomly sampled avian H1 HA sequences that represented the entire time period (1918 to 2015) of the study were also included with the swine IAV, resulting in a final data set of 7,070 H1 HA sequences.

### Phylogenetic methods, clade annotation, and clade comparisons.

From these data, a maximum likelihood tree was inferred using RAxML (v8.2.4 [54]) on the CIPRES Science Gateway (55) employing the rapid bootstrap algorithm, a general time-reversible (GTR) model of nucleotide substitution, and Γ-distributed rate variation among sites. The statistical support for individual branches was estimated by bootstrap analysis with the number of bootstrap replicates determined automatically using an extended majority-rule consensus tree criterion (56).

Using this phylogeny, we defined clades using quantifiable criteria that were applied collectively across the entire data set. Clades were defined based on sharing of a common node and monophyly, statistical support greater than 70% at the clade-defining node, and average percent pairwise nucleotide distances between and within clades of >7% and <7%, respectively, with certain minor exceptions (see Results). Given recent, relatively frequent, spillover of nonswine viruses without subsequent onward transmission in swine populations, we required a minimum of 10 viruses between 2010 and the present in a proposed clade before assigning a clade designation. Using this process, we identified three first-order lineages, seven second-order divisions, 13 third-order divisions, and eight fourth-order divisions (Fig. 1; see also Data Set S1 in the supplemental material). Sampling and sequencing in the 1900s and early 2000s were not representative of the relative abundance of different swine IAV clades (see reference 10); consequently, in Results, we restrict comments on abundance and geographical dispersion to just those data from 2010 to the present.

To validate tree topology, branch support, and the subsequent manual clade designations, we created three separate data sets by separating the 6,298 swine H1 sequences into the three first-order lineages and then randomly subsampling viruses from each second-order division. The first data set contained 750 sequences from the 1A lineage (classical swine lineage), the second data set contained 1,018 sequences from the 1B lineage (human seasonal lineage), and the third data set contained 760 sequences from the 1C lineage (Eurasian avian lineage). For each of the data sets, we inferred maximum likelihood trees according to the methods described above. In addition, we performed Bayesian analyses on each data set using mixed nucleotide models within MrBayes v 3.2.5 (57) with two parallel runs of four Markov chain Monte Carlo (MCMC) chains, each for 3 million generations, with subsampling every 100th generation. Independent replicates were conducted to determine that analyses were not trapped at local optima. We considered stationarity of molecular evolutionary parameters when effective sample sizes of >200 were reached or the potential scale reduction factor was at or near 1.0 (58). Trees prior to stationarity were burned in, and the remaining trees were used to assess posterior probabilities for nodal support. These analyses used the computational resources of the USDA-ARS computational cluster Ceres on ARS SCINet.

To quantify the within- and between-clade nucleotide distances for the H1 clade designations, the average pairwise distances (APDs) were calculated in MEGA-CC v 7.07 (59) using the *p*-distance calculation.

### Swine H1 clade classification tool.

The H1 gene classification tool is based on a bifurcating scaffold phylogenetic tree inferred using maximum likelihood from 3 to 10 representatives of each well-supported, named clade, to capture the evolutionary relationships among clades. To be included in this representative phylogeny, an H1 sequence was required to be at least 1,600 nucleotides (nt) long but was unrestricted with respect to host species. The classifier uses pplacer (60) to attach a query sequence to a branch in this tree, without reestimating the tree. Thus, the tree of representative sequences acts as a “scaffold” upon which the query sequence is placed. pplacer maximizes the likelihood of the placement by comparing the sequence of the query with the sequences in the tree, given the estimates of the evolutionary parameters underlying the inferred phylogeny. The classifier then assigns a clade to the query based on the clades represented in the local neighborhood of its placement (see Fig. S2 in the supplemental material), as follows: (i) if the query is attached to a terminal branch, then it is assigned the clade of the virus at the tip; (ii) if the query is attached to an internal branch, then it is assigned the clade of the node at the basal end of this branch. Internal nodes are assigned clades according to the rules in a parameter file. Nodes with “-like” classifications fall into internode regions joining subtrees of distinct clades. In our experience, viruses assigned “-like” classifications are often transitional, occurring prior to or during the emergence of a new clade that successfully expands onward. The “-like” designation attempts to capture the position intermediate between older and newer clades.

The classifier is written in perl and is portable, fast, and accurate. Importantly, it is adaptable readily to other clade classification tasks, because it specifies the parameters relevant to a particular application in external files. To date, it has been applied successfully to classification of avian HA/H5 sequences, according to the nomenclature of the WHO/OIE/FAO H5N1 Working Group (29), to distinguishing new pandemic 2009 H1 viruses from earlier seasonal H1 viruses in humans and other hosts, and to classifying U.S. swine H1 HA phylogenetic clades. These three applications have been implemented on IRD (30, 31).

## References

[1] Brown IH 2000 The epidemiology and evolution of influenza viruses in pigs. Vet Microbiol 74:29–46. doi:10.1016/S0378-1135(00)00164-4. 10799776

[2] Van Reeth K, Nauwynck H, Pensaert M 1996 Dual infections of feeder pigs with porcine reproductive and respiratory syndrome virus followed by porcine respiratory coronavirus or swine influenza virus: a clinical and virological study. Vet Microbiol 48:325–335. doi:10.1016/0378-1135(95)00145-X. 9054128 PMC7117459

[3] Thacker EL, Thacker BJ, Janke BH 2001 Interaction between Mycoplasma hyopneumoniae and swine influenza virus. J Clin Microbiol 39:2525–2530. doi:10.1128/JCM.39.7.2525-2530.2001. 11427564 PMC88180

[4] Garten RJ, Davis CT, Russell CA, Shu B, Lindstrom S, Balish A, Sessions WM, Xu X, Skepner E, Deyde V, Okomo-Adhiambo M, Gubareva L, Barnes J, Smith CB, Emery SL, Hillman MJ, Rivailler P, Smagala J, de Graaf M, Burke DF, Fouchier RAM, Pappas C, Alpuche-Aranda CM, López-Gatell H, Olivera H, López I, Myers CA, Faix D, Blair PJ, Yu C, Keene KM, Dotson PD, Boxrud D, Sambol AR, Abid SH, St George K, Bannerman T, Moore AL, Stringer DJ, Blevins P, Demmler-Harrison GJ, Ginsberg M, Kriner P, Waterman S, Smole S, Guevara HF, Belongia EA, Clark PA, Beatrice ST, Donis R, Katz J, Finelli L, Bridges CB, Shaw M, Jernigan DB, Uyeki TM, Smith DJ, Klimov AI, Cox NJ 2009 Antigenic and genetic characteristics of swine-origin 2009 A(H1N1) influenza viruses circulating in humans. Science 325:197–201. doi:10.1126/science.1176225. 19465683 PMC3250984

[5] Nelson MI, Vincent AL 2015 Reverse zoonosis of influenza to swine: new perspectives on the human-animal interface. Trends Microbiol 23:142–153. doi:10.1016/j.tim.2014.12.002. 25564096 PMC4348213

[6] Vijaykrishna D, Smith GJD, Pybus OG, Zhu H, Bhatt S, Poon LLM, Riley S, Bahl J, Ma SK, Cheung CL, Perera RAPM, Chen H, Shortridge KF, Webby RJ, Webster RG, Guan Y, Peiris JSM 2011 Long-term evolution and transmission dynamics of swine influenza A virus. Nature 473:519–522. doi:10.1038/nature10004. 21614079

[7] Vijaykrishna D, Poon LLM, Zhu HC, Ma SK, Li OTW, Cheung CL, Smith GJD, Peiris JSM, Guan Y 2010 Reassortment of pandemic H1N1/2009 influenza A virus in swine. Science 328:1529. doi:10.1126/science.1189132. 20558710 PMC3569847

[8] Rajão DS, Gauger PC, Anderson TK, Lewis NS, Abente EJ, Killian ML, Perez DR, Sutton TC, Zhang J, Vincent AL 2015 Novel reassortant human-like H3N2 and H3N1 influenza A viruses detected in pigs are virulent and antigenically distinct from swine viruses endemic to the United States. J Virol 89:11213–11222. doi:10.1128/JVI.01675-15. 26311895 PMC4645639

[9] Vincent AL, Lager KM, Anderson TK 2014 A brief introduction to influenza A virus in swine. Methods Mol Biol 1161:243–258. doi:10.1007/978-1-4939-0758-8_20. 24899434

[10] Vincent A, Awada L, Brown I, Chen H, Claes F, Dauphin G, Donis R, Culhane M, Hamilton K, Lewis N, Mumford E, Nguyen T, Parchariyanon S, Pasick J, Pavade G, Pereda A, Peiris M, Saito T, Swenson S, Van Reeth K, Webby R, Wong F, Ciacci-Zanella J 2014 Review of influenza A virus in swine worldwide: a call for increased surveillance and research. Zoonoses Public Health 61:4–17. doi:10.1111/zph.12049. 23556412

[11] Shope RE 1931 Swine influenza: III. Filtration experiments and etiology. J Exp Med 54:373–385. doi:10.1084/jem.54.3.373. 19869924 PMC2132000

[12] Olsen CW 2002 The emergence of novel swine influenza viruses in North America. Virus Res 85:199–210. doi:10.1016/S0168-1702(02)00027-8. 12034486

[13] Zhou NN, Senne DA, Landgraf JS, Swenson SL, Erickson G, Rossow K, Liu L, Yoon KJ, Krauss S, Webster RG 1999 Genetic reassortment of avian, swine, and human influenza A viruses in American pigs. J Virol 73:8851–8856. 10482643 10.1128/jvi.73.10.8851-8856.1999 PMC112910

[14] Karasin AI, Landgraf J, Swenson S, Erickson G, Goyal S, Woodruff M, Scherba G, Anderson G, Olsen CW 2002 Genetic characterization of H1N2 influenza A viruses isolated from pigs throughout the United States. J Clin Microbiol 40:1073–1079. doi:10.1128/JCM.40.3.1073-1079.2002. 11880444 PMC120269

[15] Lewis NS, Russell CA, Langat P, Anderson TK, Berger K, Bielejec F, Burke DF, Dudas G, Fonville JM, Fouchier RA, Kellam P, Koel BF, Lemey P, Nguyen T, Nuansrichy B, Peiris JM, Saito T, Simon G, Skepner E, Takemae N, ESNIP3 Consortium, Webby RJ, Van Reeth K, Brookes SM, Larsen L, Watson SJ, Brown IH, Vincent AL 2016 The global antigenic diversity of swine influenza A viruses. eLife 5:e01914. doi:10.7554/eLife.12217. PMC4846380 27113719

[16] Smith GJD, Vijaykrishna D, Bahl J, Lycett SJ, Worobey M, Pybus OG, Ma SK, Cheung CL, Raghwani J, Bhatt S, Peiris JSM, Guan Y, Rambaut A 2009 Origins and evolutionary genomics of the 2009 swine-origin H1N1 influenza A epidemic. Nature 459:1122–1125. doi:10.1038/nature08182. 19516283

[17] Guan Y, Shortridge KF, Krauss S, Li PH, Kawaoka Y, Webster RG 1996 Emergence of avian H1N1 influenza viruses in pigs in China. J Virol 70:8041–8046. 8892928 10.1128/jvi.70.11.8041-8046.1996 PMC190877

[18] de Jong JC, van Nieuwstadt AP, Kimman TG, Loeffen WL, Bestebroer TM, Bijlsma K, Verweij C, Osterhaus AD, Class EC 1999 Antigenic drift in swine influenza H3 haemagglutinins with implications for vaccination policy. Vaccine 17:1321–1328. doi:10.1016/S0264-410X(98)00392-2. 10195767

[19] Castrucci MR, Donatelli I, Sidoli L, Barigazzi G, Kawaoka Y, Webster RG 1993 Genetic reassortment between avian and human influenza A viruses in Italian pigs. Virology 193:503–506. doi:10.1006/viro.1993.1155. 8438586

[20] Brown IH, Chakraverty P, Harris PA, Alexander DJ 1995 Disease outbreaks in pigs in Great Britain due to an influenza A virus of H1N2 subtype. Vet Rec 136:328–329. doi:10.1136/vr.136.13.328. 7541591

[21] Lorusso A, Vincent AL, Harland ML, Alt D, Bayles DO, Swenson SL, Gramer MR, Russell CA, Smith DJ, Lager KM, Lewis NS 2011 Genetic and antigenic characterization of H1 influenza viruses from United States swine from 2008. J Gen Virol 92:919–930. doi:10.1099/vir.0.027557-0. 21177926 PMC3133703

[22] Vincent AL, Ma W, Lager KM, Gramer MR, Richt JA, Janke BH 2009 Characterization of a newly emerged genetic cluster of H1N1 and H1N2 swine influenza virus in the United States. Virus Genes 39:176–185. doi:10.1007/s11262-009-0386-6. 19597980

[23] Nelson MI, Schaefer R, Gava D, Cantão ME, Ciacci-Zanella JR 2015 Influenza A viruses of human origin in swine, Brazil. Emerg Infect Dis 21:1339–1347. doi:10.3201/eid2108.141891. 26196759 PMC4517702

[24] Anderson TK, Campbell BA, Nelson MI, Lewis NS, Janas-Martindale A, Killian ML, Vincent AL 2015 Characterization of co-circulating swine influenza A viruses in North America and the identification of a novel H1 genetic clade with antigenic significance. Virus Res 201:24–31. doi:10.1016/j.virusres.2015.02.009. 25701742

[25] Anderson TK, Nelson MI, Kitikoon P, Swenson SL, Korslund JA, Vincent AL 2013 Population dynamics of cocirculating swine influenza A viruses in the United States from 2009 to 2012. Influenza Other Respir Viruses 7(Suppl 4):42–51. doi:10.1111/irv.12193. PMC5655888 24224819

[26] Watson SJ, Langat P, Reid SM, Lam TT-Y, Cotten M, Kelly M, Van Reeth K, Qiu Y, Simon G, Bonin E, Foni E, Chiapponi C, Larsen L, Hjulsager C, Markowska-Daniel I, Urbaniak K, Dürrwald R, Schlegel M, Huovilainen A, Davidson I, Dán Á, Loeffen W, Edwards S, Bublot M, Vila T, Maldonado J, Valls L, ESNIP3 Consortium, Brown IH, Pybus OG, Kellam P 2015 Molecular epidemiology and evolution of influenza viruses circulating within European swine between 2009 and 2013. J Virol 89:9920–9931. doi:10.1128/JVI.00840-15. 26202246 PMC4577897

[27] Shortridge KF, Webster RG, Butterfield WK, Campbell CH 1977 Persistence of Hong Kong influenza virus variants in pigs. Science 196:1454–1455. doi:10.1126/science.867041. 867041

[28] Peiris JSM, Guan Y, Ghose P, Markwell D, Krauss S, Webster RG, Shortridge KF 2001 Co-circulation of avian H9N2 and human H3N2 viruses in pigs in southern China. Int Congr Ser 1219:195–200. doi:10.1016/S0531-5131(01)00667-7.

[29] WHO/OIE/FAO H5N1 Evolution Working Group 2008 Toward a unified nomenclature system for highly pathogenic avian influenza virus (H5N1). Emerg Infect Dis 14:e1. doi:10.3201/eid1407.071681. PMC2600337 18598616

[30] Squires RB, Noronha J, Hunt V, García-Sastre A, Macken C, Baumgarth N, Suarez D, Pickett BE, Zhang Y, Larsen CN, Ramsey A, Zhou L, Zaremba S, Kumar S, Deitrich J, Klem E, Scheuermann RH 2012 Influenza research database: an integrated bioinformatics resource for influenza research and surveillance. Influenza Other Respir Viruses 6:404–416. doi:10.1111/j.1750-2659.2011.00331.x. 22260278 PMC3345175

[31] Zhang Y, Aevermann BD, Anderson TK, Burke DF, Dauphin G, Gu Z, He S, Kumar S, Larsen CN, Lee AJ, Li X, Macken C, Mahaffey C, Pickett BE, Reardon B, Smith T, Stewart L, Suloway C, Sun G, Tong L, Vincent AL, Walters B, Zaremba S, Zhao H, Zhou L, Zmasek C, Klem EB, Scheuermann RH 26 9 2016 Influenza Research Database: an integrated bioinformatics resource for influenza virus research. Nucleic Acids Res doi:10.1093/nar/gkw857. PMC5210613 27679478

[32] Grgić H, Costa M, Friendship RM, Carman S, Nagy É, Poljak Z 2015 Genetic characterization of H1N1 and H1N2 influenza A viruses circulating in Ontario pigs in 2012. PLoS One 10:e0127840. doi:10.1371/journal.pone.0127840. 26030614 PMC4452332

[33] Detmer SE, Patnayak DP, Jiang Y, Gramer MR, Goyal SM 2011 Detection of influenza A virus in porcine oral fluid samples. J Vet Diagn Invest 23:241–247. doi:10.1177/104063871102300207. 21398442

[34] Simon G, Larsen LE, Dürrwald R, Foni E, Harder T, Van Reeth K, Markowska-Daniel I, Reid SM, Dán A, Maldonado J, Huovilainen A, Billinis C, Davidson I, Agüero M, Vila T, Hervé S, Breum SØ, Chiapponi C, Urbaniak K, Kyriakis CS, ESNIP3 Consortium, Brown IH, Loeffen W 2014 European surveillance network for influenza in pigs: surveillance programs, diagnostic tools and swine influenza virus subtypes identified in 14 European countries from 2010 to 2013. PLoS One 9:e115815. doi:10.1371/journal.pone.0115815. 25542013 PMC4277368

[35] Nelson MI, Viboud C, Vincent AL, Culhane MR, Detmer SE, Wentworth DE, Rambaut A, Suchard MA, Holmes EC, Lemey P 2015 Global migration of influenza A viruses in swine. Nat Commun 6:6696. doi:10.1038/ncomms7696. 25813399 PMC4380236

[36] Nelson M, Culhane MR, Rovira A, Torremorell M, Guerrero P, Norambuena J 2015 Novel human-like influenza A viruses circulate in swine in Mexico and Chile. PLoS Curr 7:ecurrents.outbreaks.c8b3207c9bad98474eca3013fa933ca6. doi:10.1371/currents.outbreaks.c8b3207c9bad98474eca3013fa933ca6. 26345598 PMC4551470

[37] Zell R, Scholtissek C, Ludwig S 2013 Genetics, evolution, and the zoonotic capacity of European swine influenza viruses. Curr Top Microbiol Immunol 370:29–55. doi:10.1007/82_2012_267. 23011571

[38] Nelson MI, Stratton J, Killian ML, Janas-Martindale A, Vincent AL 2015 Continual reintroduction of human pandemic H1N1 influenza A viruses into swine in the United States, 2009 to 2014. J Virol 89:6218–6226. doi:10.1128/JVI.00459-15. 25833052 PMC4474294

[39] Rajão DS, Anderson TK, Gauger PC, Vincent AL 2014 Pathogenesis and vaccination of influenza A virus in swine. Curr Top Microbiol Immunol 385:307–326. doi:10.1007/82_2014_391. 25033752

[40] Van Reeth K, Ma W 2012 Swine influenza virus vaccines: to change or not to change—that’s the question. Curr Top Microbiol Immunol 370:173–200. doi:10.1007/82_2012_266. 22976350

[41] Anderson TK, Laegreid WW, Cerutti F, Osorio FA, Nelson EA, Christopher-Hennings J, Goldberg TL 2012 Ranking viruses: measures of positional importance within networks define core viruses for rational polyvalent vaccine development. Bioinformatics 28:1624–1632. doi:10.1093/bioinformatics/bts181. 22495748

[42] Van Reeth K, Labarque G, De Clercq S, Pensaert M 2001 Efficacy of vaccination of pigs with different H1N1 swine influenza viruses using a recent challenge strain and different parameters of protection. Vaccine 19:4479–4486. doi:10.1016/S0264-410X(01)00206-7. 11483274

[43] Loving CL, Lager KM, Vincent AL, Brockmeier SL, Gauger PC, Anderson TK, Kitikoon P, Perez DR, Kehrli ME 2013 Efficacy in pigs of inactivated and live attenuated influenza virus vaccines against infection and transmission of an emerging H3N2 similar to the 2011–2012 H3N2v. J Virol 87:9895–9903. doi:10.1128/JVI.01038-13. 23824815 PMC3754103

[44] Kyriakis CS, Gramer MR, Barbé F, Van Doorsselaere J, Van Reeth K 2010 Efficacy of commercial swine influenza vaccines against challenge with a recent European H1N1 field isolate. Vet Microbiol 144:67–74. doi:10.1016/j.vetmic.2009.12.039. 20116942

[45] Luoh SM, McGregor MW, Hinshaw VS 1992 Hemagglutinin mutations related to antigenic variation in H1 swine influenza viruses. J Virol 66:1066–1073. 1731091 10.1128/jvi.66.2.1066-1073.1992 PMC240810

[46] Both GW, Shi CH, Kilbourne ED 1983 Hemagglutinin of swine influenza virus: a single amino acid change pleiotropically affects viral antigenicity and replication. Proc Natl Acad Sci U S A 80:6996–7000. doi:10.1073/pnas.80.22.6996. 6580621 PMC390113

[47] Allerson M, Deen J, Detmer SE, Gramer MR, Joo HS, Romagosa A, Torremorell M 2013 The impact of maternally derived immunity on influenza A virus transmission in neonatal pig populations. Vaccine 31:500–505. doi:10.1016/j.vaccine.2012.11.023. 23174202 PMC3534892

[48] Loeffen WLA, Heinen PP, Bianchi ATJ, Hunneman WA, Verheijden JHM 2003 Effect of maternally derived antibodies on the clinical signs and immune response in pigs after primary and secondary infection with an influenza H1N1 virus. Vet Immunol Immunopathol 92:23–35. doi:10.1016/S0165-2427(03)00019-9. 12628761

[49] Gauger PC, Vincent AL, Loving CL, Lager KM, Janke BH, Kehrli ME, Roth JA 2011 Enhanced pneumonia and disease in pigs vaccinated with an inactivated human-like (δ-cluster) H1N2 vaccine and challenged with pandemic 2009 H1N1 influenza virus. Vaccine 29:2712–2719. doi:10.1016/j.vaccine.2011.01.082. 21310191

[50] Katoh K, Misawa K, Kuma K-I, Miyata T 2002 MAFFT: a novel method for rapid multiple sequence alignment based on fast Fourier transform. Nucleic Acids Res 30:3059–3066. doi:10.1093/nar/gkf436. 12136088 PMC135756

[51] Katoh K, Standley DM 2013 MAFFT multiple sequence alignment software version 7: improvements in performance and usability. Mol Biol Evol 30:772–780. doi:10.1093/molbev/mst010. 23329690 PMC3603318

[52] Maddison WP, Maddison DR 2015 Mesquite: a modular system for evolutionary analysis. Version 2.75, 2011 http://mesquiteproject.org.

[53] Schloss PD, Westcott SL, Ryabin T, Hall JR, Hartmann M, Hollister EB, Lesniewski RA, Oakley BB, Parks DH, Robinson CJ, Sahl JW, Stres B, Thallinger GG, Van Horn DJ, Weber CF 2009 Introducing mothur: open-source, platform-independent, community-supported software for describing and comparing microbial communities. Appl Environ Microbiol 75:7537–7541. doi:10.1128/AEM.01541-09. 19801464 PMC2786419

[54] Stamatakis A 2014 RAxML version 8: a tool for phylogenetic analysis and post-analysis of large phylogenies. Bioinformatics 30:1312–1313. doi:10.1093/bioinformatics/btu033. 24451623 PMC3998144

[55] Miller MA, Pfeiffer W, Schwartz T 2010 Creating the CIPRES Science Gateway for inference of large phylogenetic trees, p 1–8. *In* Proceedings of the Gateway Computing Environments Workshop (GCE). IEEE, New York, NY.

[56] Pattengale ND, Alipour M, Bininda-Emonds ORP, Moret BME, Stamatakis A 2010 How many bootstrap replicates are necessary? J Comput Biol 17:337–354. doi:10.1089/cmb.2009.0179. 20377449

[57] Ronquist F, Huelsenbeck JP 2003 MrBayes 3: Bayesian phylogenetic inference under mixed models. Bioinformatics 19:1572–1574. doi:10.1093/bioinformatics/btg180. 12912839

[58] Gelman A, Rubin DB 1992 Inference from iterative simulation using multiple sequences. Stat Sci 7:457–472. doi:10.1214/ss/1177011136.

[59] Kumar S, Stecher G, Peterson D, Tamura K 2012 MEGA-CC: computing core of molecular evolutionary genetics analysis program for automated and iterative data analysis. Bioinformatics 28:2685–2686. doi:10.1093/bioinformatics/bts507. 22923298 PMC3467750

[60] Matsen FA, Kodner RB, Armbrust EV 2010 pplacer: linear time maximum-likelihood and Bayesian phylogenetic placement of sequences onto a fixed reference tree. BMC Bioinformatics 11:538. doi:10.1186/1471-2105-11-538. 21034504 PMC3098090

[61] Marozin S, Gregory V, Cameron K, Bennett M, Valette M, Aymard M, Foni E, Barigazzi G, Lin Y, Hay A 2002 Antigenic and genetic diversity among swine influenza A H1N1 and H1N2 viruses in Europe. J Gen Virol 83:735–745. doi:10.1099/0022-1317-83-4-735. 11907321

