# Supplementary figures and images for: A Phylogeny-Based Global Nomenclature System and Automated Annotation Tool for H1 Hemagglutinin Genes from Swine Influenza A Viruses
Source: mSphere. 2016 Dec 14;1(6):e00275-16. doi: 10.1128/mSphere.00275-16 (PMC5156671; doi:10.1128/mSphere.00275-16)

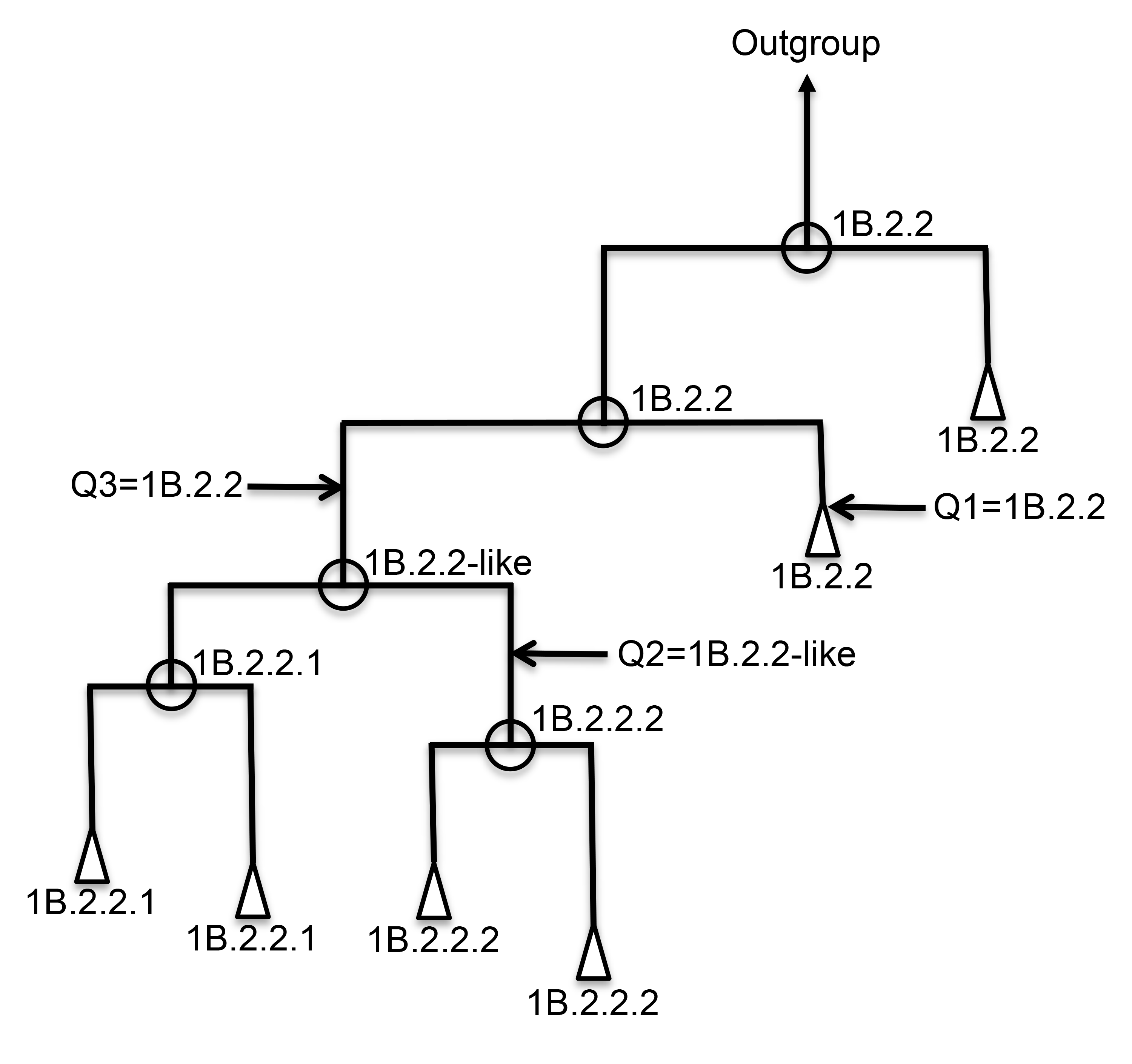

Supplement: Figure S2 [file sph006162204sf2.tif]
